# Protective Effect Against Toxoplasmosis in *BALB/c* Mice Vaccinated With Recombinant *Toxoplasma gondii* MIF, CDPK3, and 14-3-3 Protein Cocktail Vaccine

**DOI:** 10.3389/fimmu.2021.755792

**Published:** 2021-12-22

**Authors:** Fang Liu, Minmin Wu, Jie Wang, Hongyang Wen, Ran An, Haijian Cai, Li Yu, Jilong Shen, Lijian Chen, Jian Du

**Affiliations:** ^1^ Department of Biochemistry and Molecular Biology, School of Basic Medical Sciences, Anhui Medical University, Hefei, China; ^2^ The Research Center for Infectious Diseases, School of Basic Medical Sciences, Anhui Medical University, Hefei, China; ^3^ The Provincial Key Laboratory of Zoonoses of High Institutions of Anhui, Anhui Medical University, Hefei, China; ^4^ The Key Laboratory of Microbiology and Parasitology of Anhui Province, Anhui Medical University, Hefei, China; ^5^ Department of Anesthesiology, The First Affiliated Hospital of Anhui Medical University, Hefei, China

**Keywords:** *Toxoplasma gondii*, TgMIF, TgCDPK3, Tg14-3-3, cocktail vaccine, protective efficacy

## Abstract

*Toxoplasma gondii* can infect almost all endotherm organisms including humans and cause life-threatening toxoplasmosis in immunocompromised individuals, which leads to serious public health problems. Developing an excellent vaccine against this disease is impending. In present study, we formulated a cocktail protein vaccine including the TgMIF, TgCDPK3, and Tg14-3-3 proteins, which play critical roles in *T. gondii* infection. The recombinant protein vaccines were constructed and assessed by vaccination in *BALB/c* mice. We organized the mice in various protein combination groups of vaccines, and all mice were immunized with corresponding proteins at 0, 2, and 4 weeks. The specific protective effects of the vaccines on mice against *T. gondii* were analyzed by the mensuration of cytokines, serum antibodies, splenocyte proliferation assay, survival time, and parasite cyst burden of mice after the challenge. The study indicated that mice immunized with all three multicomponent proteins vaccine triggered a strong immune response with highest levels of IFN-γ production and IgG antibody compared with the other two protein combinations and controls. Moreover, there was an increase in IL-4 production and antigen-specific lymphocyte proliferation. The parasite cysts were significantly reduced (resulting in an 82.7% reduction), and survival time was longer in immunized mice with three multicomponent proteins compared with the other groups of mice. The enhanced humoral and cell-mediated immunity indicated that the protein cocktail vaccine containing three antigens provided effective protection for mice. These results indicated that recombinant TgMIF, TgCDPK3, and Tg14-3-3 multicomponent proteins were potential candidates for vaccine against toxoplasmosis.

## Introduction

*Toxoplasma gondii* (*T. gondii*) is an obligate intracellular protozoan parasite. The life cycle of *T. gondii* is very complex and comprises two specific phases, thus, sexual and asexual cycles. The sexual cycle occurs only in cats, the definitive host. The asexual cycle occurs in other warm-blooded animals including humans. The parasite exists in two distinct forms: tachyzoites (the rapidly dividing form observed in the acute phase of infection) and bradyzoites (the slowly growing form observed in tissue cysts). For most people, *Toxoplasma gondii* causes asymptomatic infections and continues to live in the host. However, among immunocompromised individuals and pregnant women, *T. gondii* may be an important opportunistic pathogen, which can cause serious diseases such as encephalitis, miscarriage, and stillbirth. Moreover, toxoplasmosis could cause severe abortion and neonatal death in domestic animals, which could pose enormous economic impact (1). Unfortunately, current forms of medication for toxoplasmosis (atovaquone, sulfadiazine, and pyrimethamine) only target the acute stage, while no specific drugs are effective against the chronic stage (2, 3). To date, only one commercial vaccine is developed to control *Toxoplasma*-associated abortion in sheep, which comprises live tachyzoites of the S48 “incomplete” strain of *T. gondii*. Moreover, the available drugs have severe side effects to the host, and reactivation may occur at any time (4). As such, under the present scenario, developing an effective vaccine against toxoplasmosis is of vital importance (5). *T. gondii* possesses a complex life cycle with diversity of form and presents a plurality of antigenic epitopes, which varies widely among different strains. Increasing evidence suggest that vaccination with simplex stage antigens only lead to partial protection (6). It is, therefore, difficult to achieve a recombinant vaccine with complete protection against complex *T. gondii* infection with only one immunodominant antigen. It was reported that multiprotein vaccination would better protect mice from toxoplasmosis than single-protein vaccines (7). The purpose of our study was to determine the protective efficacy of a multiprotein vaccine against acute or chronic *T. gondii* infection. TgMIF, TgCDPK3, and Tg14-3-3, which played important roles in *T. gondii* infection, were selected to be configured into a cocktail protein vaccine.

Macrophage migration inhibitory factor (MIF) is a cytokine that plays a central role in immune and inflammatory responses (8). It was first described as a factor produced by lymphocytes associated with inhibition of random macrophage migrations during delayed hypersensitivity response (8). MIF homologs of different vertebrates appear universally to be involved in innate and adaptive immune responses, affect cell migration, proinflammatory cytokine secretion, and cell differentiation or morphogenesis (9). Significantly, homologs of MIF have been described in many parasite species including *Plasmodium*, *Leishmania*, *Brugia*, *Clostridium*, and *Toxoplasma*, and it has been implied that these proteins facilitate the manipulation of the host immune response during infection (10–13). *Toxoplasma gondii* MIF (TgMIF) is a 13-kDa secretory protein which lacks oxidoreductase activity but exhibits tautomerase activity. TgMIF is expressed in both tachyzoite and bradyzoite forms of the parasite (14). Our previous research revealed that TgMIF is a good vaccine candidate due to its excellent antigenic index and surface probability (15).

The distinct family of calcium-dependent protein kinases (CDPKs) of *Toxoplasma* contains fourteen CDPKs which play substantial roles in the *Toxoplasma* life cycle including replication, egress, gliding motility, and invasion. TgCDPK3 is a serine/threonine kinase belonging to a large family of CDPKs, and is found in plants and ciliates, but absent in humans (16). TgCDPK3 is localized to the parasite periphery in intracellular and extracellular parasites and partially to the apical end of the intracellular parasite (17). TgCDPK3 is a key enzyme to control calcium-dependent permeabilization in parasitophorous vacuole membrane during parasite egress and tissue cyst formation. The size of TgCDPK3 is approximately 60 kDa, with stable protein properties, good hydrophilicity, and sufficient structural diversity. It is a good antigen and a stable protein with an instability index of 32.03. Six B-cell epitopes have been found in the protein sequence, which can better present antigen particles by antigen presenting cells (18, 19). Hence, TgCDPK3 protein possesses the extensive potential to be applied for developing a vaccine against *T. gondii*.

14-3-3 proteins are a family of ubiquitously expressed proteins of all eukaryotic cells. As dimerized adapter proteins, they are involved in the regulation of diverse cellular processes including cytoskeleton organization, cell migration, apoptosis, and cell cycle control (20). In *T. gondii*, Tg14-3-3 protein is found in the oocyst stage and the tachyzoite stage and is located in the cytoplasm, parasitophorous vacuole membrane, and excreted/secreted antigen (ESA) (21). Studies have reported that Tg14-3-3 in tachyzoite ESA can stimulate the immune system and induce a cell-mediated as well as antibody-dependent protective response (22). In addition, Tg14-3-3 can induce hypermigration of parasitized microglia and dendritic cells, suggesting that Tg14-3-3 may play a role in the dissemination of parasites to the target host tissues (23). Moreover, previous research revealed Tg14-3-3 as a novel target for intracellular pathogen that acts by hijacking the migratory properties of the host cell to disseminate. Immunization of recombinant 14-3-3 protein could stimulate the host immune system to produce an intense response. Therefore, 14-3-3 protein was identified as a potential vaccine candidate against toxoplasmosis (24).

Previous studies have investigated cocktail antigen vaccines to prevent *T. gondii* infection. However, the majority of studies focused on the DNA cocktail vaccines. Until now, there have been several studies on cocktail antigens of recombinant proteins against toxoplasmosis. Among them, some studies were about mixed vaccines of the same class of proteins, such as MIC-mixed, GRA-mixed, or ROP-mixed vaccines (25–28). Recent studies showed that some of these vaccines were effective in the prevention of acute toxoplasmosis (28–30), while others were effective at preventing chronic toxoplasmosis (25, 31–33). Jongert et al. (34) identified the chimeric protein rEC2 encoding antigenic fragments of surface-associated proteins MIC2, MIC3, and SAG1, and they demonstrated that the vaccine had a preventive effect on chronic toxoplasmosis. Since TgMIF, TgCDPK3, and Tg14-3-3 are expressed at different stages of the parasite, we hypothesized that a combination of these three proteins may be a promising vaccine against toxoplasmosis.

## Materials and Methods

### Ethics Statement

The protocol for the animal experiments for this study was approved by the Ethics Committee of the Animal Experiments of Anhui Medical University (permit number 20180016). The study was carried out in strict accordance with the recommendations of the Guide for the Care and Use of Laboratory Animals of Anhui Medical University. All efforts were made to minimize animal suffering of these studies.

### Animals and Parasites

Female *BALB/c* mice, aged 6–8 weeks were maintained in a SPF standard condition and freely took food and water. The tachyzoites of *Toxoplasma gondii* RH strain (type I) were maintained in HFF cells, and the bradyzoites of PRU strain (type II) were obtained from orally infected brain of Kunming mice in this study.

### Preparation of rTgMIF, rTgCDPK3, and rTg14-3-3

The fragments of TgMIF (GenBank™ ID XP_002368429.1) and TgCDPK3 (GenBank™ ID XP_002370358.1) genes were amplified by RT-PCR from the *T. gondii* (ME49 strain) tachyzoites RNA. Similarly, Tg14-3-3 (GenBank™ ID AB012775) gene was amplified from *T. gondii* (RH strain) RNA. Gene fragments of TgMIF, TgCDPK3, and Tg14-3-3 were then inserted into pET28a vector (Novagen, Madison, WI, USA), and the sequences were verified by DNA sequencing. The recombinant pET28a/TgMIF, pET28a/TgCDPK3, and pET28a/Tg14-3-3 plasmids were transferred into BL21 host bacteria cells (Transgen, Beijing, China). Single colonies were cultured in LB substrate supplemented with antibiotics at 37°C at 230 rpm. Each culture was then inoculated to 500 ml fresh LB medium containing antibiotics to cultivate largely, which was incubated at 30°C for 5.5 h with constant shaking at 230 rpm followed by addition of 0.1 mM isopropyl-beta-d-thiogalactopyranoside (IPTG). The recombinant cells were harvested *via* centrifugation, and the obtained pellets were resuspended in lysis buffer (pH 8.0). TgMIF, TgCDPK3, and Tg14-3-3 bacteria pellets were then disrupted by sonication for 8 min at 4 s interval on ice, respectively. Next, the lysate was centrifuged at 12,000×*g* for 20 min to obtain the supernatant. All purification processes were performed at 4°C. To purify target proteins, the supernatants were applied onto 1 ml Ni^2+^-NTA agarose by gentle rotation for 1 h at 4°C (Qiagen, Hilden, Germany), respectively. After binding, the target proteins were washed and eluted with buffer containing imidazole in different concentrations. rTgMIF, rTgCDPK3, and rTg14-3-3 were detected by Western blotting and Coomassie brilliant blue staining.

### Western Blotting

The eluted rTgMIF, rTgCDPK3, rTg14-3-3, and rTgROP18 were separated using 12% SDS-PAGE gels. After electrophoresis, proteins were electroblotted onto a nitrocellulose membrane. The membranes were blocked in 5% BSA for 1 h at room temperature and incubated with the rabbit primary antibody of anti-TgMIF, anti-TgCDPK3, and anti-Tg14-3-3 overnight at 4°C, severally. Next, the nitrocellulose membranes were treated with secondary antibodies (Proteintech, Wuhan, China) at room temperature for 1 h, and target proteins were detected by ECL (Tanon, Shanghai, China).

### rTgMIF, rTgCDPK3, and rTg14-3-3 Immunization

Six groups (G1–G6) of 6- to 8-week-old *BALB/*c mice (*n* = 40 each group) were used for immunization experiments. We designed two control groups: blank and PBS control (G1 and G2) and four immunization groups. The immunization doses were determined through previous research (25, 28, 35–38). G3 was immunized with proteins containing two antigens (rTgMIF and rTgCDPK3), G4 was immunized with proteins containing two antigens (rTgMIF and rTg14-3-3), G5 was immunized with proteins containing two antigens (rTgCDPK3 and rTg14-3-3), and G6 was immunized with proteins containing three antigens (rTgMIF, rTgCDPK3, and rTg14-3-3). **Table 1** shows the details of vaccination program in different groups. The mice of G1 were blank control and G2 were treated with 100 μl sterile PBS *via* intraperitoneal injection. All immunization proteins were mixed with complete Freund’s adjuvant (CFA) at first immunization and Freund’s incomplete adjuvant (IFA) for booster injections with the same doses. Blood samples were collected and stored at −80°C.

**Table 1:** The detail of vaccination regimens.

Group	Immunization protocol	Content	Volume	Administration
G1	Blank control	–	–	–
G2	PBS	–	100 μl	Intramuscular
G3	MIF+CDPK3	6 μg + 6 μg	100 μl	Intramuscular
G4	MIF+14-3-3	6 μg + 6 μg	100 μl	Intramuscular
G5	CDPK3+14-3-3	6 μg + 6 μg	100 μl	Intramuscular
G6	MIF+CDPK3+14-3-3	4 μg + 4 μg + 4 μg	100 μl	Intramuscular

### Antibody Detection

Blood samples of mice in each group were collected by tail-bleeding on days 0, 14, and 28 and 2 weeks after the final immunization (on day 42), then centrifuged at 5,000×*g* for 10 min, and the sera isolated were stored at −80°C. Briefly, microtiter plates were coated with corresponding immune proteins (10 μg/ml) overnight at 4°C. Nonspecific binding was blocked with 1% BSA at 37°C for 1 h and washed with PBST containing 0.05% Tween 20 (pH 7.4). The IgG endpoint antibody titers were estimated with serial dilutions (7 points, starting at 1:800) of each sample. Serum was attenuated in 0.1% BSA-PBST kept at 37°C for 1 h. After washing, 100 μl second antimouse antibodies HRP-IgG, HRP-IgG1, or HRP-IgG2a (Proteintech, China) diluted in PBST was added in each well and incubated for 1 h at 37°C. Wells were then washed 5 times with PBST. Finally, the immune complexes were developed by incubation with TMB (100 μl/well) for 30 min. The reaction was stopped by adding terminate liquid to each well. The absorbance was detected at OD_450 nm_, and all assays were performed in triplicate.

### Spleen Cell Proliferation

Two weeks after final booster immunization, nine mice were euthanized in each group; the spleen cells were isolated and processed to generate splenocyte suspension through a mesh sieve. Next, splenocyte were resuspended in RPMI 1640 medium supplemented with 10% fetal bovine serum (FBS). Cell were cultured in 96-well and adjusted to a final concentration of 2 × 10^5^ cells per well. Splenocyte were stimulated with corresponding proteins respectively or medium alone for 72 h with 5% CO_2_ at 37°C. Splenocyte proliferative activity was determined by Cell Counting Kit-8 (Beyotime, Haimen, China), and the stimulation index was calculated as the ratio of the average OD_450_ value of stimulation cells wells to the average OD_450_ value of unstimulated cell wells with medium.

### Production of Cytokines

Splenocytes harvested from nine mice in each group were seeded into 24-well plates at 1.5 × 10^6^ cells/well. Spleen cells from G1 to G2 mice were cultured with medium alone. Spleen cells from G3 mice were stimulated with rTgMIF and rTgCDPK3 (5 μg/ml of each protein); G4 were stimulated with rTgMIF and rTg14-3-3 (5 μg/ml of each protein); G5 were stimulated with rTgCDPK3 and rTg14-3-3 (5 μg/ml of each protein), and G6 were stimulated with rTgMIF, rTgCDPK3, and rTg14-3-3 (3.3 μg/ml of each protein). Culture supernatants were collected, and the levels of IFN-γ, IL-2, IL-10, and IL-4 were measured using a commercial enzyme-linked immunosorbent assay (ELISA) kit (Shanghai Enzyme-linked Biotechnology Co. Ltd., Shanghai, China) according to the manufacturer’s recommendations. Data from three independent experiments were used in the analysis.

### Challenge Infection

Two weeks after the third immunization, 10 mice in each group were challenged intraperitoneally with 1 × 10^3^ tachyzoites of *T. gondii* RH strain diluted in 100 μl PBS and observed daily for an additional 30 days; deaths were recorded as they occurred. Two weeks after the last immunization, three mice per group were infected orally with 20 cysts of the PRU strain. One month after infection, brains of mice from each group were homogenized in 1 ml PBS. The number of cysts per brain was determined by three samples of 10 μl aliquots of each homogenized brain under an optical microscope.

### Statistical Analysis

Statistical analysis and graphic work were performed using GraphPad Prism 5.0. The difference in antibody levels in all mice groups was compared using two-way ANOVA. Survival rate was determined by the Kaplan-Meier approach and compared with the log-rank test. Other data were compared by one-way ANOVA. The results were considered statistically significant if *p* < 0.05.

## Result

### Recombinant TgMIF, TgCDPK3, and Tg14-3-3 Expression and Purification

**Figure 1:**
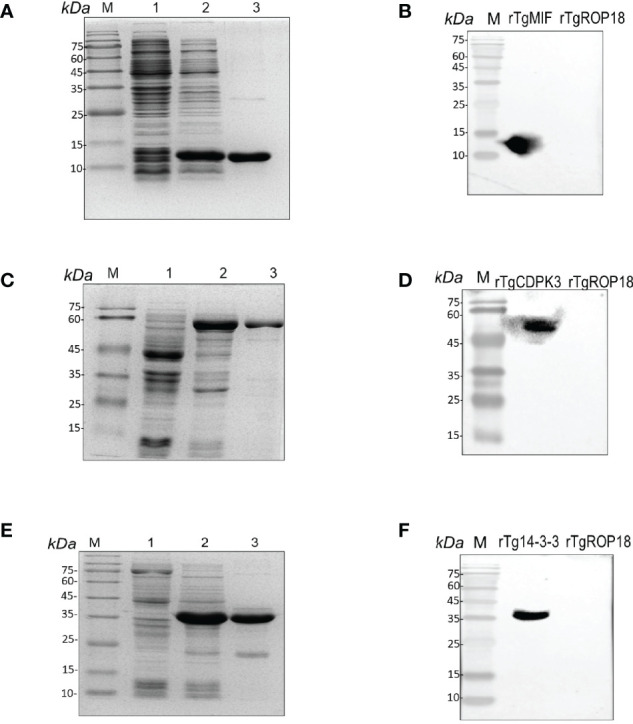
Identification of rTgMIFr, TgCDPK3, and rTg14-3-3 protein expression. **(A)** Purified rTgMIF size was stained by Coomassie blue approximately 13 kDa. M, protein marker. Lanes 1–2, lysate of bacteria transferred with pET-28a-TgMIF before and after IPTG treatment. Lane 3, the purified rTgMIF. **(B)** Purified rTgMIF was detected by Western blotting. **(C)** Purified rTgCDPK3 was stained by Coomassie blue approximately 59 kDa. M, protein marker. Lanes 1–2, lysate of bacteria transferred with pET-28a-TgCDPK3 before and after IPTG treatment. Lane 3, the purified rTgCDPK3. **(D)** Purified rTgCDPK3 was detected by Western blotting. **(E)** Purified rTg14-3-3 was stained by Coomassie blue approximately 35 kDa. M, protein marker. Lanes 1–2, lysate of bacteria transferred with pET-28a-Tg14-3-3 before and after IPTG treatment. Lane 3, the purified rTg14-3-3. **(F)** Purified rTg14-3-3 was detected by Western blotting.

The hydrophilic plot, flexible region, antigen index, and surface probability of rTgMIF, rTgCDPK3, and rTg14-3-3 proteins were analyzed by DNAStar (**Supplementary Figure S1**). The three proteins were then expressed in *Escherichia coli* and purified by prepacked Ni^2+^-charged Sepharose columns. SDS-PAGE detected the rTgMIF, rTgCDPK3, and rTg14-3-3, respectively. The results revealed that these purified proteins had the molecular weight of approximately 13, 59, and 35 kDa, which corresponded with the predicted sizes (**Figures 1A, C, E**). Furthermore, the specificity of prokaryotic expression of rTgMIF, rTgCDPK3, and rTg14-3-3 proteins were identified by the polyclonal antibody against TgMIF, TgCDPK3, and Tg14-3-3. In addition, another purified recombinant parasite protein, rTgROP18 was not detected by the antibodies of the above three proteins (**Figures 1B, D, F**). Thus, these results indicated that these three recombinant *Toxoplasma* proteins had been successfully expressed and purified.

### Evaluation of Humoral Responses

**Figure 2:**
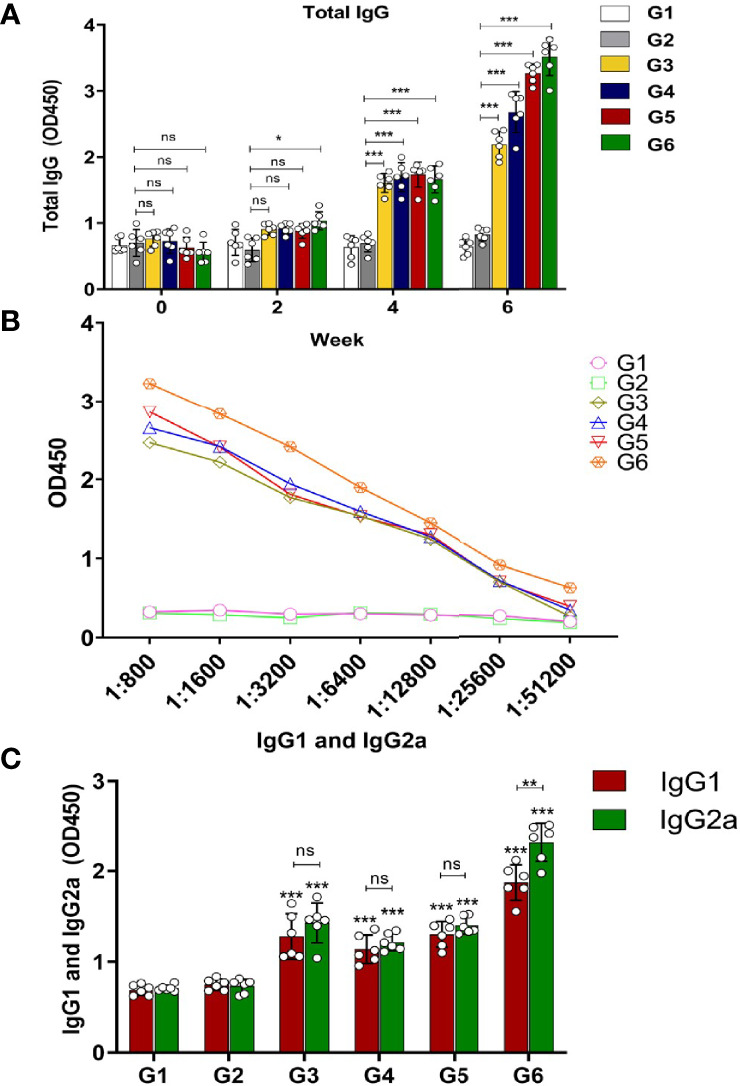
Determination of specific humoral response in the immunized mice. **(A)** Serum was collected to detect total IgG antibodies at preinjection and 2, 4, and 6 weeks after immunization. **(B)** Evaluation of endpoint IgG antibody titers with serial dilutions (up to 1:51,200). **(C)** Detection of IgG1 and IgG2a antibodies in immunized mice groups 2 weeks after the last immunization. Experimental groups include G1 blank control mice; G2 mice received PBS alone; G3 mice received proteins containing two antigens (rTgMIF and rTgCDPK3); G4 mice received proteins containing two antigens (rTgMIF and rTg14-3-3); G5 mice received proteins containing two antigens (rTgCDPK3 and rTg14-3-3); G6 mice received three proteins containing (rTgMIF, rTgCDPK3, and rTg14-3-3). The results were expressed as mean ± SD (*n* = 6). ^***^p < 0.001, ^**^p < 0.01, *p < 0.05, ns, no significant.

To evaluate whether immunization protocol (**Supplementary Figure S2**) with these three recombinant *Toxoplasma* proteins could elicit humoral responses, serum samples of six groups were collected on days 0, 14, and 28 postimmunization and 2 weeks after the final immunization (on day 42), and IgG antibodies of mice in all groups were analyzed by ELISA. The results revealed that there was no significant difference in IgG antibody level in the control groups (G1 and G2). Total IgG in mice of G3, G4, and G5 increased moderately with continuous immunization and reached a peak at 6 weeks after the last booster immunization. Among them, the G6-immunized mice had the highest immune response (**Figure 2A**). Along with the increased times and kinds of proteins used in immunization, the IgG titer became higher and higher. The IgG endpoint antibody titers in the serum of immunized mice were detected up to 1:51,200 dilutions (**Figure 2B**). To further clarify whether Th1/Th2 immune-type responses were elicited in the immunized mice, the levels of antibody subclass (IgG1 and IgG2a) isotypes at 2 weeks after the last immunization were analyzed. As shown in **Figure 2C**, IgG1 and IgG2a antibody levels notably increased in the sera of G3–G5 mice compared with the control groups, suggesting that two protein combinations were able to elicit Th1/Th2 immune response (*p* < 0.001). Moreover, both IgG1 and IgG2a were apparently increased in G6, suggesting three protein combination also stimulated a mixed Th1/Th2 immune response. However, IgG2a was slightly higher than IgG1 in the G6 group, indicating that the immune response was biased towards Th1 immunity (*p* < 0.01) (**Figure 2C**). In general, G6 (rTgMIF, rTgCDPK3, and rTg14-3-3) immunization group induced the highest immune response, with a slight bias toward the Th1-type response.

### Evaluation of Lymphocyte Proliferation

**Figure 3:**
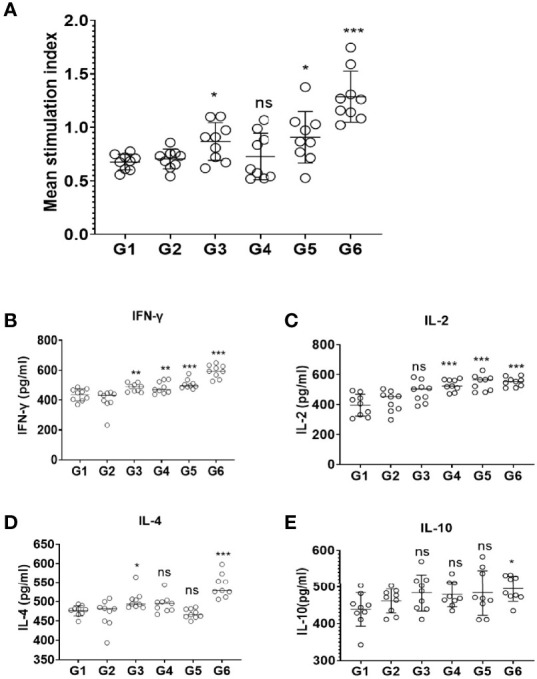
Splenocyte proliferation and cytokines production after vaccination. **(A)** Spleen lymphocytes were harvested from vaccinated mice 2 weeks after the last injection. The CCK-8 assay was used to detect proliferative response of spleen lymphocytes. The results are expressed as the stimulation index (SI) ± SD (*n* = 9). Cytokines secreted by mice splenocytes after immunizing with different cocktail vaccines. **(B)** IFN-γ. **(C)** IL-2. **(D)** IL-4. **(E)** IL-10. Experimental groups include G1 blank control mice; G2 mice received PBS alone; G3 mice received proteins containing two antigens (rTgMIF and rTgCDPK3); G4 mice received proteins containing two antigens (rTgMIF and rTg14-3-3); G5 mice received proteins containing two antigens (rTgCDPK3 and rTg14-3-3); G6 mice received three proteins containing (rTgMIF, rTgCDPK3, and rTg14-3-3). Each bar represents the mean ± SD (*n* = 9). ^***^p < 0.001; ^**^ *p* < 0.01; ^*^ *p* < 0.05; ns, not significant.

Splenocytes from the mice of immunization and control groups were cultured and the proliferation was observed by Cell Counting Kit-8 assay. The results revealed that there was no significant difference between the control groups. However, splenic lymphocytes of mice from G3 and G5 groups were slightly increased (*p* < 0.05); G6 (rTgMIF, rTgCDPK3, and rTg14-3-3) mixed recombinant proteins significantly enhance proliferative response compared with PBS group (*p* < 0.001) (**Figure 3A**). These results suggest that mice immunized with a mixture of rTgMIF, rTgCDPK3, and rTg14-3-3 induced more intense antigen-specific lymphocyte response compared with other groups.

### Expression of Cytokines

To detect the production and types of cytokines, the supernatant of spleen cells stimulated by proteins in different combinations were collected and tested by ELISA. The data showed that the levels of IFN-γ of G3-G6 mice were increased compared with the PBS group. G4 and G5 mice injected with two recombinant proteins induced Th1-type immune responses and evoked high levels of IFN-γ. Furthermore, G6-immunized mice generated Th1/Th2-type immune response which was defined by high levels of IFN-γ and IL-2 and slightly increased IL-4 and IL-10 levels (**Figures 3B**–**E**). These findings indicate that immunization with the three proteins cocktail vaccine could induce a mixed Th1/Th2 protective immune response. Moreover, the three-protein-combination vaccine caused high level of IFN-γ and IL-2 instead of IL-4 and IL-10, indicating that Th1-type immune was the dominant response.

### Protection of Vaccinated Mice

**Figure 4:**
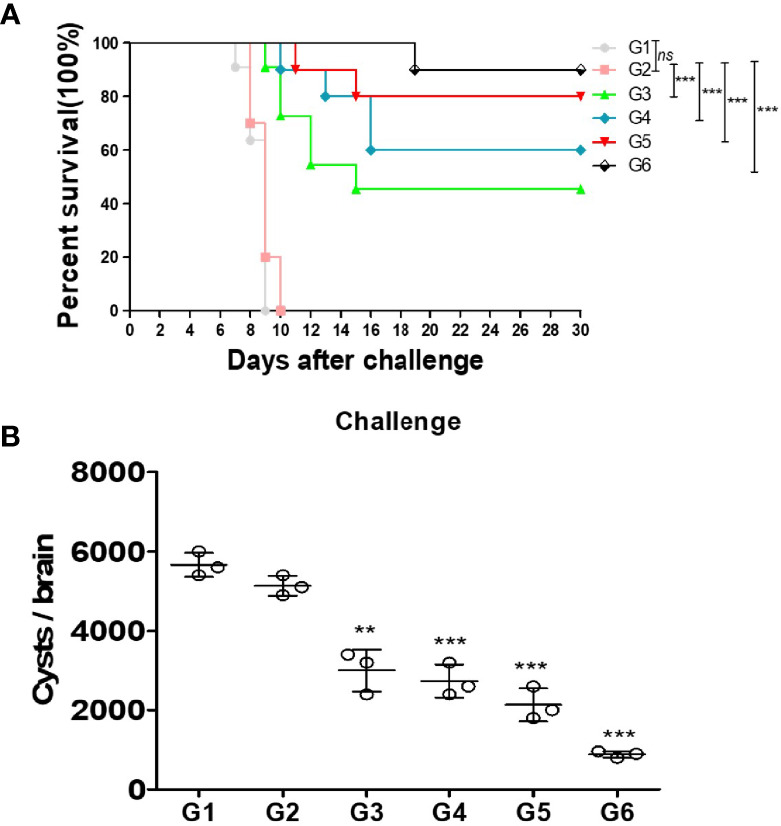
Protection of vaccines immunized mice against acute and chronic *Toxoplasma gondii* infection. **(A)** Fourteen days after the final immunization, all mice were injected with 1 × 10^3^ tachyzoites of RH strain. The results are expressed as the mean ± SD (*n* = 10). The survival time of mice immunized with recombination proteins were significantly longer than controls (blank or PBS). ^***^p < 0.001; ns, not significant. Gehan–Breslow–Wilcoxon tests. **(B)** Fourteen days after the last immunization, these mice were challenged with 20 PRU strain cysts by intragastric administration. All samples were analyzed for three times. After a month, the mean number of cysts of each group (three mice) was harvested from the brain of mice. Experimental groups include G1 blank control mice; G2 mice received PBS alone; G3 mice received proteins containing two antigens (rTgMIF and rTgCDPK3); G4 mice received proteins containing two antigens (rTgMIF and rTg14-3-3); G5 mice received proteins containing two antigens (rTgCDPK3 and rTg14-3-3); G6 mice received three proteins containing (rTgMIF, rTgCDPK3 and rTg14-3-3). The results are expressed as the mean ± SD (*n* = 3). ^***^ *p* < 0.001; ^**^ *p* < 0.01.

To examine the effective protection of the different cocktail vaccines against *T. gondii* acute infection, all groups were infected with 1 × 10^3^ RH strain tachyzoites by intraperitoneal injection. All mice from the blank group or immunized with PBS control died within 10 days after RH strain tachyzoite challenge. Notably, prolonged survival time was observed in mice immunized with mixtures of different proteins. Particularly, the survival time of G6 group mice was the highest among the groups with 90% survival rate (**Figure 4A**). These results indicate that these proteins conferred partial protection against *T. gondii* acute infection. Nevertheless, the G6 mice immunized with a mixture of three recombinant proteins showed the strongest protection in acute infection. Furthermore, the protein vaccines were assessed against *T. gondii* chronic infection. Mice were infected with 20 PRU cysts for 1 month *via* intragastric administration. The tissue cysts lodged in the brains of G1–G6 mice were counted. As shown, the amount of brain cysts in G3–G6 mice were significantly reduced compared with that in the PBS group, indicating that the vaccines provided partial protection in mice against *T. gondii* chronic infection. Particularly, the cocktail of rTgMIF, rTgCDPK3, and rTg14-3-3, resulted in a significant decrease in the number of brain cysts about 82.7% (**Figure 4B**). These results showed that the compound of rTgMIF, rTgCDPK3, and rTg14-3-3 provided more effective protection in mice than the other groups against *T. gondii* in both acute and chronic challenges.

## Discussion

Toxoplasmosis is a severe disease threatening human health, which is linked to mental disorders and may affect human behavior personality and other phenotypic traits. *T. gondii* had a wide distribution and high prevalence in many areas, and more than one-third of the world’s population was infected. Thus, the development of an effective vaccine anti-*Toxoplasma* would be of great significance in preventing infection in immunocompromised individuals and economic losses in the livestock industry (39). The types of vaccines include inactivated vaccines, live-attenuated vaccines, DNA vaccines, protein vaccines, epitope vaccines, and live vector-based vaccines (7, 40, 41). Many studies have tested the vaccines designed by surface antigens (SAGs), microneme antigens (MICs), rhoptry antigens (ROPs), dense granule antigens (GRAs), and other antigens secreted by tachyzoites and bradyzoites in animal models. Recombinant protein vaccines are still the main force against acute and chronic infections of *T. gondii* by being safe and efficient, which have a great potential for the prevention or eradication of the disease (42).

Different antigens that constituted a compound are considered to produce a stronger immunity response against *T. gondii* infection on account of containing more epitopes, in theory, which is superior to a single antigen (7). A previous study demonstrated that mice vaccinated with cocktail DNA plasmids of TgGRA24, TgGRA25, and TgMIC6 induced better protective immunity against acute and chronic *T. gondii* infection in the mice model (43). The purpose of this study was to evaluate the protective efficacy of protein multicomponent vaccines against acute and chronic toxoplasmosis. Three well-characterized antigens which play key roles in host–parasite interactions, were selected, including TgMIF, TgCDPK3, and Tg14-3-3 (16, 19, 20, 23, 44, 45). Our study revealed that cocktail protein vaccine is a partial but effective method against acute or chronic toxoplasma infection, compared with vaccines only designed in single or two antigens (15, 46). The study indicated that there is an increase in survival time and reduction of brain cysts in immunized mice, as well as enhanced humoral and cellular immune responses compared with the control mice groups or infected mice immunized with any two proteins.

Mechanisms of immunity to toxoplasmosis dependent on Th1 cell-mediated immunity is driven by the production of high levels of IL-12 and IFN-γ (47). Innate immune cells migrate to the site of infection where they detect the parasite, mainly *via* Toll-like receptors (TLRs), and secrete IL-12, to stimulate CD4^+^ and CD8^+^ T cells, and natural killer (NK) cells to produce IFN-γ (48). IFN-γ is an irreplaceable cytokine that limit the parasite’s proliferation and the progression of infection. Once infection is resolved, some of these cells survive in the body to form immunologic memory ready to fight the toxoplasma faster and more efficiently, when a second encounter occurs. Vaccination takes advantage of this potential of adaptive immunity. The principle of vaccination is that exposure to a small sample of a disease-causing microorganism—or to a part or portion of it—teaches the immune system to rapidly recognize the menace and to create memory that enables the body to fight the real pathogen efficiently during a later encounter. Vaccination basically mimics a natural infection without causing a disease (49). Several components of host immune responses are set in motion following vaccination. Important vaccine-induced immune effectors are antibodies produced by B lymphocytes. These antibodies are capable of recognizing and specifically binding to a toxin or a pathogen (or to a portion representative of it). Antibody binding interferes with pathogen entry into host cells or facilitates uptake and elimination of the pathogen by other immune cells. Other important immune effectors are cytotoxic CD8^+^ T cells that may limit the spread of infectious agents by recognizing and killing infected cells or by secreting specific cytokines. The generation and maintenance of both B- and CD8^+^ T-cell responses are supported by growth factors and signals provided by CD4^+^ T helper (Th) lymphocytes. Effector CD4^+^ Th cells were initially subdivided into T-helper 1 (Th1) or T-helper 2 (Th2) subsets depending on their main cytokine production (IFN-γ or IL-4), respectively. A good vaccine could induce a mixed Th1/Th2 immune response, with a slight bias toward the Th1-type response (49–51). The present study showed that G4 and G5 mice injected with two recombinant proteins induced Th1-type immune responses and evoked high levels of IFN-γ. Moreover, G6 mice injected with three proteins generated Th1/Th2-type concomitant immune response defined by high levels of IL-2 and IFN-γ and slight increase of IL-4 and IL-10. As the proinflammatory cytokine, IFN-γ and IL-2 were released largely to limit *T. gondii* during different infection phases; they are considered to be indicator for triggering of CD4^+^ Th1 and CD8^+^ cytotoxic T cells (52). IL-4 plays an important role in the early stage of acute infection with *T. gondii*, and it helps to prevent acute infection to some extent. As the “anti-inflammatory” cytokine of Th2 type, IL-10 could inhibit IFN-γ-mediated macrophage killing activity to balance inflammatory reaction. These findings indicated that immunization with the three-protein cocktail vaccine could induce a mixed Th1/Th2 protective immune response. The vaccine caused high level of IFN-γ and IL-2 instead of IL-4 and IL-10, indicating that Th1-type immune was the dominant response.

It is well-known that anti-*T. gondii* IgG exert a crucial role in preventing attachment between *T. gondii* and host cell and collaborating with macrophages for killing parasites (53). Moreover, IgG antibody was indispensable in controlling cyst reactivation during chronic infection. In this study, the level of anti-*T. gondii*-specific IgG from mice immunized with recombinant protein compound increased with successive immunization. The level of IgG in immunized mice showed an obvious increase in comparison with the controls. Moreover, the levels of anti-*T. gondii* IgG antibodies in G6 were higher than G3–G5 immunized by the two-protein cocktail vaccines. It was verified by further experiments on subclasses of IgG that IgG2a was predominant in G3–G5, indicating the two-protein cocktail elicited humoral immunoreaction mediated by Th1. While both IgG1 and IgG2a distinctly increased with more IgG2a than IgG1 in G6, suggesting three-protein cocktail induced a mixed Th1/Th2 immune response, biased predominantly the Th1 response. Particularly, G6 displayed the most prominent increase of IgG, IgG1, or IgG2a, which emphasized again that immune responses can be dramatically enhanced by protein immunization with multiple antigens (36, 54).

To further evaluate the protective efficacy of recombinant protein mixture vaccine, mice in each group were injected with RH tachyzoites in enterocoelia or instilled PRU cysts *via* intragastric delivery, respectively. So far, there are no vaccines that could provide a complete protection against the highly virulent *T. gondii* RH strain infected by intraperitoneal challenge (55). In our study, all mice immunized with blank or PBS control died within 10 days after RH tachyzoite challenge. Nevertheless, an effective and considerable protection was acquired in mice immunized with the protein vaccine. All groups immunized with the recombinant protein vaccine showed longer survival time after being infected with RH tachyzoites. Furthermore, mice in G6 had the highest survival rate (more than 80%, *p* < 0.001). Therefore, the three recombinant proteins produced a considerable protection, which could partially prevent mice from *T. gondii* fatal infection. To assess the resistance to chronic infection, mice were challenged with PRU cysts, a moderately virulent *T. gondii* strain. The data indicated that cocktail protein vaccines decreased the number of brain cysts of G3–G6, compared with the control groups. Particularly, mice in G6 reached a least number of brain cysts, which caused 82.7% reduction (*p* < 0.001). Until now, the only successful vaccine against *Toxoplasma gondii in vivo* is Toxovax^®^, which protects sheep from congenital infection. However, it is not known whether Toxovax^®^ vaccine has any effect on the formation of tissue cysts (56). *T. gondii* has a multistage life cycle, and the functional proteins expressed at each stage are different. These three proteins we selected cover the different developmental stages and subcellular localization, and all of them play an irreplaceable role in the life regulation of *T. gondii*. Multiantigen vaccines designed by proteins or peptides from different periods of the parasite, seem more powerful than single-antigen or double-antigen vaccines. When the three proteins are used as immune antigens at the same time, the immune response can be stimulated through various mechanisms, which can produce a stronger protective effect.

In recent years, several approaches have been used to develop novel toxoplasmosis vaccines. The types of nanoparticle-based vaccines, exosome-based vaccines, carbohydrate-based vaccines, and live-attenuated vaccines based on gene editing have been developed to elicit protective immunity against *T. gondii* infection. The abovementioned emerging vaccines have great advantages in preparing vaccines and inducing immune responses. First, nanoparticles (NPs) could maintain the integrity of the antigen, prolonging its systemic circulation time and enhancing the chance of immune cell recognition (57). Second, exosomes can transfer mRNA, miRNA, and proteins to perform intercellular communication between cells, which can maintain immune homeostasis. Third, carbohydrates on the pathogen surface are recognized by the host innate immune receptors, leading to the production of antiglycan antibodies (58). The effectiveness of a vaccine is influenced by its composition, where vaccines are composed of an antigen and an adjuvant. Adjuvant is required to enhance the immune response, which have been widely used, such as Alum, complete Freund’s adjuvant, MF59-Novartis, and AS03 (59–61). However, these adjuvants also result in unwanted systemic and local side effects; only a few adjuvants are approved for use in humans. Pathogen-associated molecular patterns (PAMPs) and TLR agonists are at the forefront of adjuvant development. PAMPs can activate specific pattern recognition receptors (PRRs) and increase immunogenicity without systemic toxicity (61). TLRs are well characterized, and their administration can elicit a strong Th1 response. Application of novel and more rational adjuvant could influence the development of safer and more effective vaccines.

In summary, our research demonstrated that recombinant proteins TgMIF, TgCDPK3, and Tg14-3-3 cocktail vaccine successfully elicited a strong humoral and cellular immune response in mice, which possessed a certain resistance against *T. gondii* infection at different stages. The multiplexing protein cocktail vaccine also induced strong and balanced Th1- and Th2-type responses, which provided partial but effective protection against both chronic and acute *Toxoplasma* infection. In our future studies, we will combine TgMIF, TgCDPK3, and Tg14-3-3 with PAMPs, TLRs, or other novel adjuvants to design different strategies against toxoplasmosis, which may immensely improve the safety and efficacy of vaccines.

## Data Availability Statement

The raw data supporting the conclusions of this article will be made available by the authors, without undue reservation.

## Ethics Statement

The animal study was reviewed and approved by the Ethics Committee of the Animal Experiments of Anhui Medical University (permit number 20180016).

## Author Contributions

JD and LC: conceived and designed the experiments. FL: methodology, formal analysis, and writing—review and editing. MW and HW: performed the experiments and analyzed the data. JW: methodology and formal analysis. RA: methodology and formal analysis. HC: formal analysis. LY: formal analysis. JS: formal analysis. All authors read and approved the final version of the manuscript.

## Funding

This work was supported by the National Natural Science Foundation of China (No. 82072300, No. 81871674, and No. 81902084).

## Conflict of Interest

The authors declare that the research was conducted in the absence of any commercial or financial relationships that could be construed as a potential conflict of interest.

## Publisher’s Note

All claims expressed in this article are solely those of the authors and do not necessarily represent those of their affiliated organizations, or those of the publisher, the editors and the reviewers. Any product that may be evaluated in this article, or claim that may be made by its manufacturer, is not guaranteed or endorsed by the publisher.

## Supplementary Material

Supplementary Figure S1 Linear-B cell epitopes of TgMIF, TgCDPK3 and Tg14-3-3 predicted by DNASTAR in hydrophilicity plot, flexible regions, antigenic index, and surface probability rules.

Supplementary Figure S2 Experimental protocol for immunization, collection for spleen cells and blood samples and challenge infection. **(A)** In the first experimental procedure mice were intraperitoneally injected with corresponding proteins emulsified in Freund’s complete adjuvant on day 1. Mice were boosted at the same dose and regimen on day 15 and 29, now emulsified in Freund’s incomplete adjuvant. Two weeks after the last injection, spleen cells were collected to assess spleen cells proliferation and cytokines. Blood samples were harvested by tail-bleeding on day 0, 14, 28 and 42 then centrifuged at 5000×g for 10 min, and the sera isolated were stored at -80°C. **(B)** Two weeks after the last immunization, mice were intraperitoneally injected 1× 10³ tachyzoites in 100 μL PBS and observed daily for an additional 30 days, deaths were recorded as they occurred. **(C)** Two weeks after the last immunization, the mice were orally infected with 20 cysts of the PRU strain. One month after infection, brains of mice from each group were homogenized in 1ml PBS. The number of cysts per brain was determined by three samples of 10 μL aliquots of each homogenized brain under an optical microscope.
