# Supplementary figures and images for: Protective Effect Against Toxoplasmosis in BALB/c Mice Vaccinated With Recombinant Toxoplasma gondii MIF, CDPK3, and 14-3-3 Protein Cocktail Vaccine
Source: Front Immunol. 2021 Dec 22;12:755792. doi: 10.3389/fimmu.2021.755792 (PMC8727341; doi:10.3389/fimmu.2021.755792)

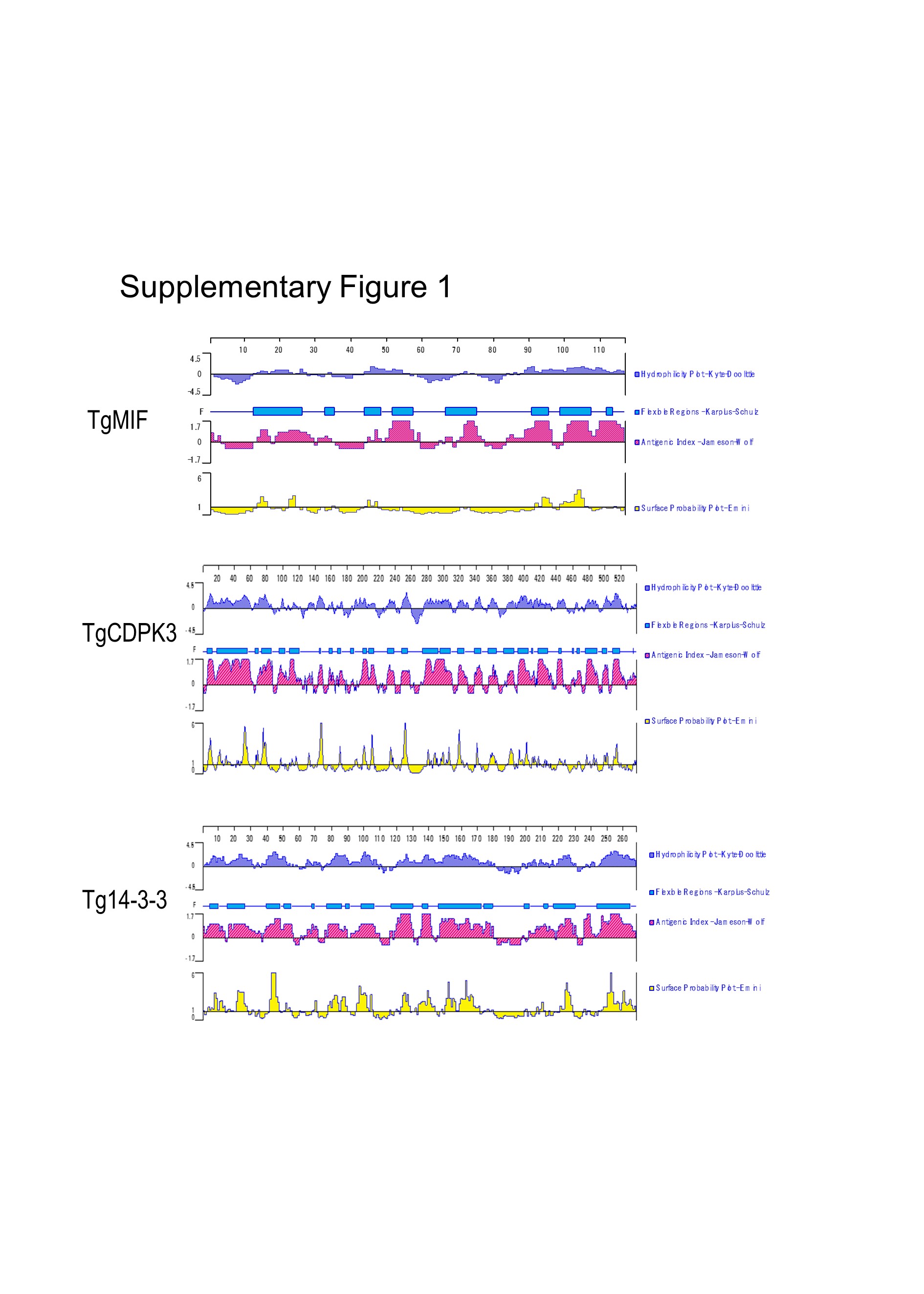

Supplement: Supplementary Figure S1 — Linear-B cell epitopes of TgMIF, TgCDPK3 and Tg14-3-3 predicted by DNASTAR in hydrophilicity plot, flexible regions, antigenic index, and surface probability rules. [file Image_1.jpeg]

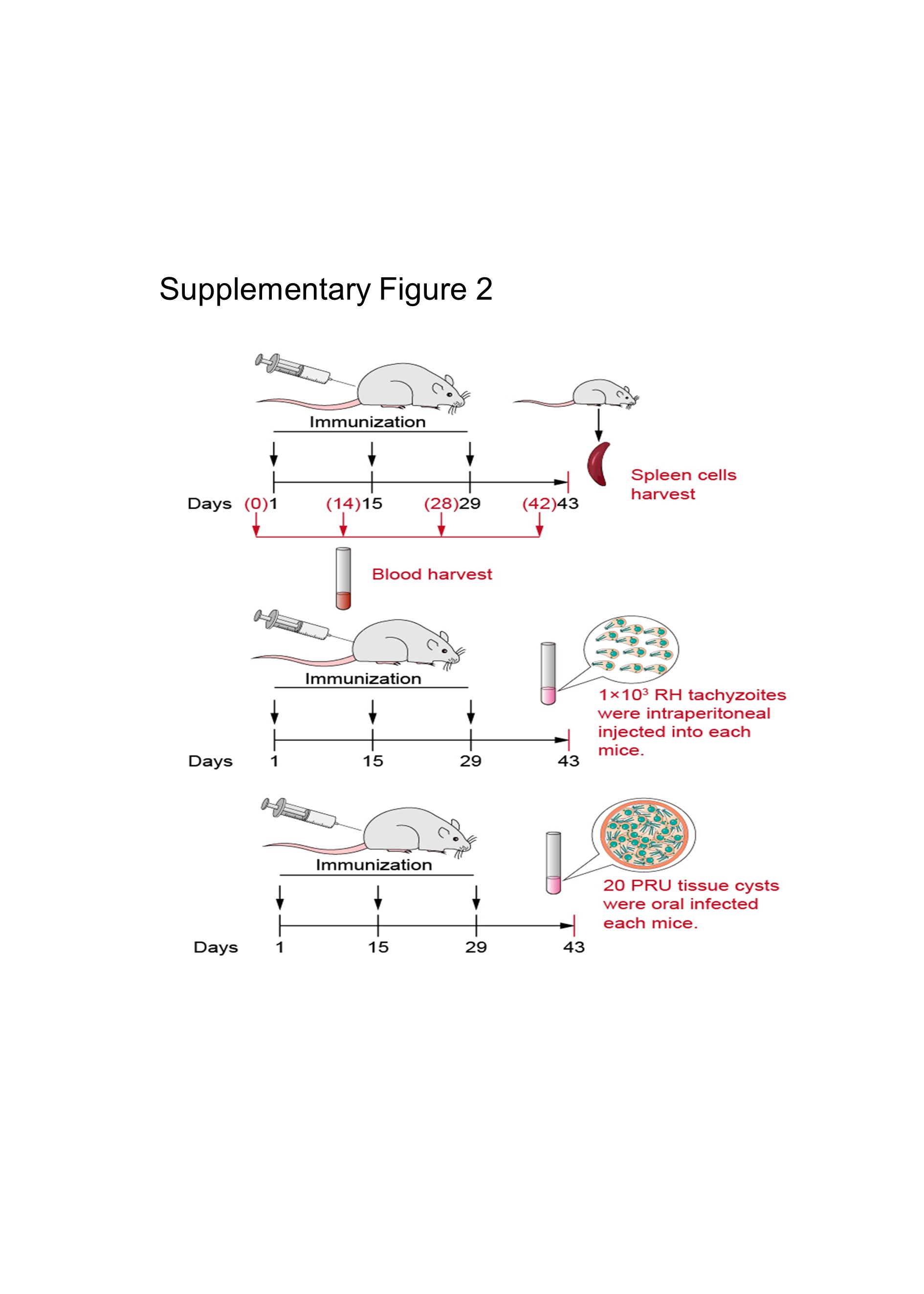

Supplement: Supplementary Figure S2 — Experimental protocol for immunization, collection for spleen cells and blood samples and challenge infection. (A) In the first experimental procedure mice were intraperitoneally injected with corresponding proteins emulsified in Freund’s complete adjuvant on day 1. Mice were boosted at the same dose and regimen on day 15 and 29, now emulsified in Freund’s incomplete adjuvant. Two weeks after the last injection, spleen cells were collected to assess spleen cells proliferation and cytokines. Blood samples were harvested by tail-bleeding on day 0, 14, 28 and 42 then centrifuged at 5000×g for 10 min, and the sera isolated were stored at -80°C. (B) Two weeks after the last immunization, mice were intraperitoneally injected 1× 10³ tachyzoites in 100 μL PBS and observed daily for an additional 30 days, deaths were recorded as they occurred. (C) Two weeks after the last immunization, the mice were orally infected with 20 cysts of the PRU strain. One month after infection, brains of mice from each group were homogenized in 1ml PBS. The number of cysts per brain was determined by three samples of 10 μL aliquots of each homogenized brain under an optical microscope. [file Image_2.jpeg]
